# A framework for the assessment of the spatial and temporal patterns of threatened coastal delphinids

**DOI:** 10.1038/srep19883

**Published:** 2016-01-25

**Authors:** Jingzhen Wang, Yingting Yang, Feng Yang, Yuelin Li, Lianjie Li, Derun Lin, Tangtian He, Bo Liang, Tao Zhang, Yao Lin, Ping Li, Wenhua Liu

**Affiliations:** 1 Marine Biology Institute, Shantou University, Guangdong 515063, China; 2 College of Ocean, Qinzhou University, Guangxi 535000, China; 3 Department of Biology and Chemistry, City University of Hong Kong; 4 The Chinese University of Hong Kong.

## Abstract

The massively accelerated biodiversity loss rate in the Anthropocene calls for an efficient and effective way to identify the spatial and temporal dynamics of endangered species. To this end, we developed a useful identification framework based on a case study of locally endangered *Sousa chinensis* by combining both LEK (local ecological knowledge) evaluation and regional boat-based survey methods. Our study investigated the basic ecological information of *Sousa chinensis* in the estuaries of eastern Guangdong that had previously been neglected, which could guide the future study and conservation. Based on the statistical testing of reported spatial and temporal dolphins sighting data from fishermen and the ecological monitoring analyses, including sighting rate, site fidelity and residence time estimations, some of the current *Sousa chinensis* units are likely to be geographically isolated and critically endangered, which calls for much greater conservation efforts. Given the accelerated population extinction rate and increasing budgetary constraints, our survey pattern can be applied in a timely and economically acceptable manner to the spatial and temporal assessment of other threatened coastal delphinids, particularly when population distributions are on a large scale and traditional sampling methods are difficult to implement.

Human activities have profoundly affected marine ecosystem via direct and indirect means^1^,^2^,^3^,^4^. Habitat loss, fragmentation and spatial heterogeneity are increasingly common in the sea, resulting in the division of large, continuous habitats into small and isolated habitat patches, in which ecological processes are altered, contributing significantly (resulting in 37% of reported marine taxa extinctions) to the decline, isolation and loss of wild populations^4^,^5^,^6^,^7^,^8^,^9^,^10^. Population isolation and fragmentation diminish the connectivity among habitat patches, leading to cascading geographical isolation, the restriction of gene flow and ultimately population extinction^11^,^12^,^13^,^14^,^15^. In this context, marine mammals, which can serve as indicators or sentinels of ecosystem health^16^,^17^,^18^,^19^, are more sensitive to environment stressors than other species and are more likely to undergo population decline and local extinction^2^,^3^. Predictive models of extinction risk indicate that approximately 37% of all marine mammals are now at risk of extinction^20^. Habitat loss and fragmentation are the main (become an increasingly dominant threat over the next 150 years) threats^3^,^4^,^20^. The extinction process leaves different spatial signatures on species’ distribution patterns^21^,^22^. It is for the conservation of marine mammals to decipher the evolutionary trend of spatial isolation before extinction occurs because the knowledge of the spatial and temporal patterns of the indicator and umbrella species will allow wildlife and natural resource managers to make better spatial management plans^18^,^19^,^23^,^24^. Given that coastal dolphins, which are restricted inshore and are seriously affected by escalating anthropogenic activities^20^,^25^,^26^, are the most threatened marine mammal species, we aimed to use a case study of a coastal dolphin species to establish an effective framework to determine temporal and large-scale spatial patterns.

Humpback dolphins (genus *Sousa*) are small cetaceans that are widely distributed in the eastern Atlantic, Indian, and western Pacific Oceans (Fig. 1a)^27^,^28^,^29^,^30^. As shallow coastal waters inhabitants, they also come into particularly frequent contact with human activity and are influenced by habitat changes^31^. One paramount issue for conservation is to evaluate the evolutionary divergence and isolation, which helps to identify conservation units. Recent studies have provided a robust and clear picture of the divergence patterns showing that at least four species in genus *Sousa*, which might be driven by environmental breakdowns, are now on separated evolutionary tracks^27^,^28^. Therefore, from the perspective of species-level conservation, a “divide and conquer” strategy is recommended^28^. However, these four species may share a similar fate as that of other coastal and riverine dolphin species: being exposed to levels of human impact that may threaten local populations^26^,^32^. Given population extinctions are a more sensitive indicator of the loss of biological capital than species extinctions^33^,^34^, we argue that spatial pattern investigation and regional threat assessment at the conspecific population level are of great necessity.

The Indo-Pacific humpback dolphins (IPHBD) in China are known as Chinese white dolphins (*Sousa chinensis*). Historically distributed from the mouth of the Yangtze River to the Vietnam border to the north (Fig. 1a)^35^, the population decreased as the habitat dwindled, and now only five habitation locales are reported^31^. Previous study indicated that Xiamen (XM) and the Pearl River Estuary (PRE) are the two main habitats for IPHBDs^35^,^36^,^37^,^38^. However, very little is known about the ecology of IPHBDs between these two waters (eastern Guangdong). The deficiency may lead to an omission of IPHBD groups, which may occur around estuarine waters in the regions. Additionally, the lack of necessary information about population dynamics and spatial patterns makes it hard to determine whether the populations from the two sites are isolated or whether there are migratory corridors supplying the exchanges between these two units, hampering the effective regional risk assessment and conservation. Several genetic studies have been conducted to estimate the genetic variability and population differentiation of these two populations^39^,^40^,^41^,^42^, but no consistent conclusions have been reached, which is likely a reflection of the constraints of their sampling designs and methodology.

We hypothesized that there is a certain amount of IPHBDs, historically or currently, in the estuaries of eastern Guangdong, according to previously revealed habitat use patterns of humpback dolphins^35^,^43^,^44^,^45^. We established a pattern using local ecological knowledge (LEK) and regional boat-based field surveys to assess the spatial and temporal dynamics of IPHBDs in eastern Guangdong. In the absence of empirical datasets, first, the basic ecology information of IPHBD sightings and distribution patterns was collected using the LEK method, which has been shown to be a useful method in the spatial and temporal distribution of aquatic animals^46^,^47^,^48^. At the same time, a systematic regional vessel-based survey was conducted to gather population ecological data between 2012 and 2014. Site fidelity and residence patterns of IPHBDs in this area were estimated. Finally, by integrating the datasets, we established a framework to analyze the spatial patterns of IPHBDs in this area. The presented study provides a flexible template for regional and global efforts to identify spatial patterns of vulnerable or endangered species, to inform further basic conservation research and education and to perform coastal spatial planning and habitat management, especially when no historical and spatial field data are available.

## Results

### Fisherman interviews

#### Demographic variables and sample bias tests (DV and SB tests)

Using the fisherman interview survey, we obtained 232 validated questionnaires. All informants were male and ranged in age from 20–83 (mean 48.68 ± SE 0.826), and the years of fishing experience were from 1–60 (mean 27.15 ± SE 0.846) (Fig. 2a). Although there was inevitable spatial variation in the distribution of fisherman settlements and fishing areas, most informants reported that they spent substantial time fishing or travelling through the estuary near the harbor where they lived, where most IPHBDs tend to be densely distributed^35^. Additionally, many informants reported that “where there are dolphins aggregating, there are many fish”. We were therefore confident that our interviews obtained detailed IPHBD observation data in each location. Although there were some fluctuations (Fig. 2b) in fishermen ages and years fishing, no statistically significant differences (*df*_age_ = 11, F_age_ = 1.733, p_age_ = 0.068, *df*_fishing_ = 11, F_fishing_ = 1.576, p_fishing_ = 0.107) were detected in these 12 fishing communities. In the multiple comparisons using the Scheffe and Bonferroni methods, no significant differences were found between locations. Fisherman age and years fishing was not a predictor of IPHBD sightings. No significant correlations were found between fisherman age (years fishing) and whether they saw an IPHBD (p_age_ = 0.532; p_fishing_ = 0.15).

### Reported sighting proportion tests (RSP tests)

**Figure 1:**
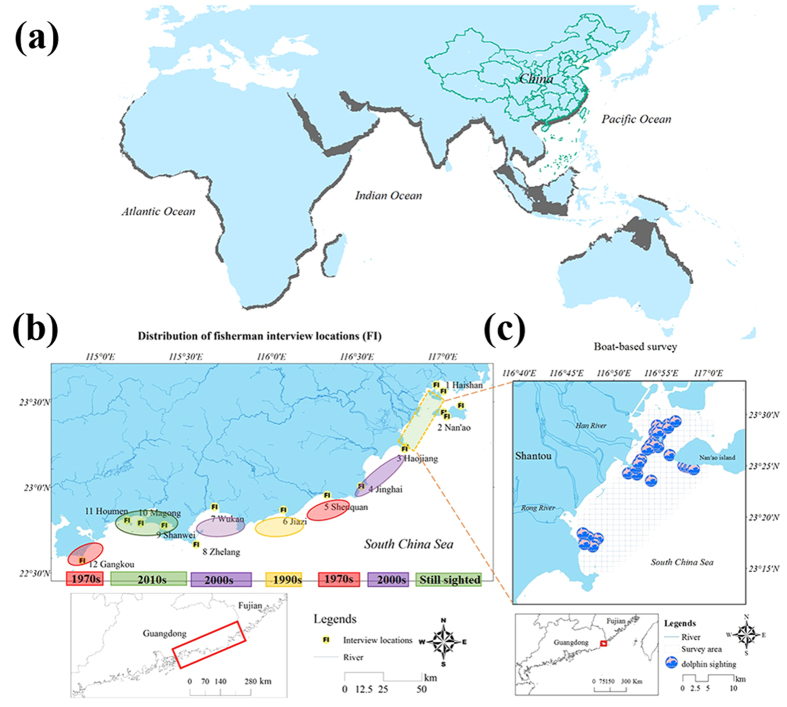
Survey maps. (**a**) The global distribution ranges of humpback dolphin (genus *Sousa*), shown as shaded regions along the coasts; (**b**) Eastern Guangdong coastline (from XM to PRE) showing the distribution of fishermen interview locations (FI), the color bar and the times under the map, which were derived from the interview data, indicate the possible IPHBD subpopulation temporal changes (the time when the distribution gap occurred); (**c**) Shantou waters, showing the boat-based survey area (grids area). Inset map underneath is a map of Guangdong and Fujian showing the location of the study area (boxed action). We created the maps with the program ArcMap of ArcGIS [10.2.2] (http://www.esri.com/software/arcgis). Map (**a**) was developed from previously reported global population distribution data of the humpback dolphin^27^,^28^,^29^,^30^.

**Figure 2:**
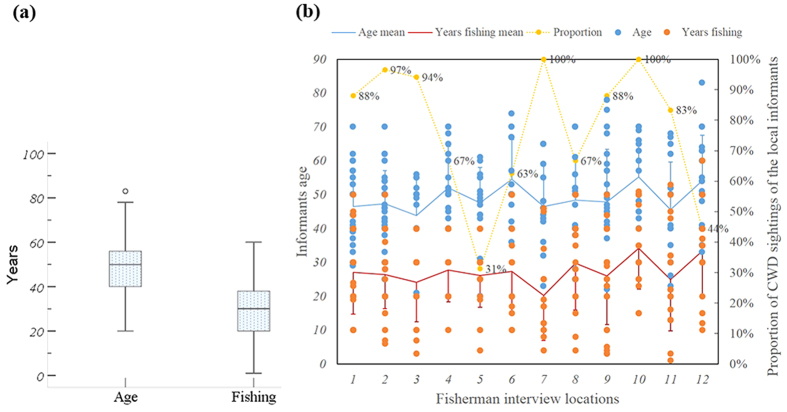
The informants’ information that we sampled in our questionnaire. (**a**) The box plots of fisherman age and years of fishing; (**b**) Distribution of age and years fishing in each surveyed location (1–12 are locations from Raoping to Gangkou town in Fig. 1b) and the proportion of IPHBD sightings of the informants.

**Figure 3:**
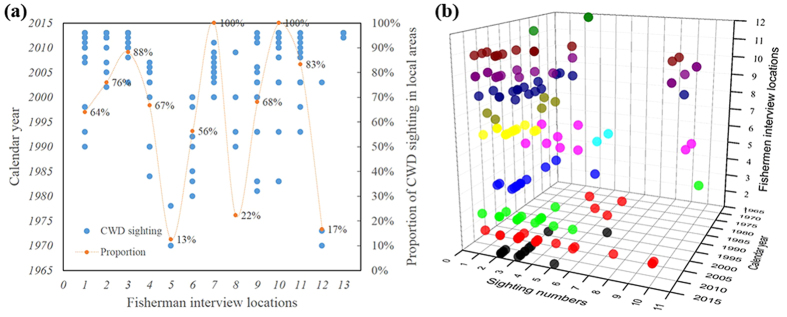
Temporal distribution of IPHBD last-sighting dates (a) and reported IPHBD group size (b) across the 12 surveyed locations (1–12 are locations from Raoping to Gangkou Town in Fig. 1b; here, 13 represents the locations where the informants saw IPHBDs outside of the study region, such as Hainan, PRE *et al.*).

Of the 232 informants, 181 reported that they had seen IPHBDs one or more times. When asked about their perceptions of changes in IPHBD sightings, 95.4% of the informants who responded this question (n = 177) considered that the IPHBD sighting rate in the present was lower than that in the past. The chi-square tests indicated that the proportions of IPHBD sightings by the local fishing communities were differently distributed across the surveyed locations (χ^2^ = 58.13, *df* = 11, p < 0.001). Only 31% of the informants in Shenquan Town and 44% informants in Gangkou Town had ever seen IPHBDs (Fig. 2b). After removing some of the responders who encountered IPHBDs in other places (e.g., Hainan, PRE, Xiamen and Shantou), the sighting rates were even lower (13% and 17%) in these two locations (Fig. 3a). In contrast, the observed values of IPHBD sighting proportions in other locations exceeded their expected frequencies (p < 0.001), indicating a spatial correlation of IPHBD sightings. In addition, we set several surveyed communities or docks in one estuary (Fig. 1b,e,g., two sites in Haishan of Raoping, three sites in Nanao of Shantou, three locations in Honghai Bay-Shanwei, Magong, and Houmen). The results of the sighting data indicated congruence, and no evidence of a significant difference was found (p = 0.374, 0.588 and 0.282).

### Latest sighting and group size tests (LS and GS tests)

The data sets indicated that the distribution of latest IPHBD sighting times also varied across the surveyed locations (Fig. 3a). Raoping, Shantou (Nanao and Haojiang on the map) and Honghai Bay (Shanwei, Magong and Houmen on the map) reported IPHBD sightings in 2013 (Fig. 3). The mean group size of reported IPHBDs in these three locations were 2.8, 3.73 and 1, respectively. In contrast, Shenquan, Zhelang and Gangkou Town had lower sighting proportions and earlier latest-sighting-dates, as well as a smaller IPHBD group size (Fig. 3). The variance analysis showed the significant geographic difference of the latest sighting records (*df* = 11, F = 9.292, p < 0.001). In the further multiple comparisons by the Scheffe test, the adjacent locations, such as Jinghai and Shenquan (p = 0.013), Jiazi and Wukan (p = 0.034), and Houmen and Gangkou Town (p = 0.005), presented significant variations, indicating different temporal change patterns of IPHBD units among the surveyed locations, whereas there were no significant differences found among Raoping and Shantou (p = 0.998) or Shanwei, Magong and Houmen (P = 0.999, 1.0, 0.984). When we tested the distribution and medians of the latest 10-year group size of IPHBD using Kruskal-Wallis test, the results also showed significant spatial differences (p < 0.001). These results indicated that the IPHBD distribution gaps likely occurred in Gangkou Town, Jiazi, Shenquan and Jinghai in the 1970 s, 1990 s, 1970 s and 2000 s, respectively (Fig. 3). The spatial and temporal distribution patterns are presented on the map (Fig. 1).

### Seasonal preference tests (SP tests)

**Figure 4:**
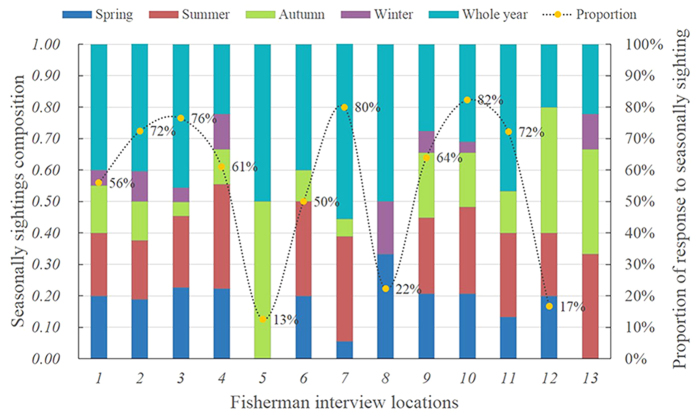
Seasonal tendency of encountering IPHBDs in each location and the proportion of responses indicating seasonal sightings (the proportion that encountered IPHBD minus the proportion that did not give an exact answer to the seasonal sighting questions) in each location.

Although there were different seasonal ratios in each surveyed location (Fig. 4), no evidence of apparent spatial variation was found in the reported IPHBD seasonal investigation by chi-square test (χ^2^ = 33.184, *df* = 48, p = 0.949). When we investigated the seasonal bias of all surveyed locations using variance analysis, significant differences were found across the five responses of seasonal questions (*df* = 4, F = 16.308, p < 0.001). In the post-hoc tests using the Siak method, the fifth answer (most of the year or no seasonal correlation) was significantly more common than other answers (p ≤ 0.001). In contrast, the proportion of reports in the winter season lagged far behind the other seasons (p < 0.05) and no significant variation was found between spring and autumn (p = 1.00). We attributed the lower proportion of winter preference to lower fishermen survey efforts in the winter due to circumstances such as bad weather conditions^49^.

### Vessel-based survey

#### Photo identification (PI)

**Figure 5:**
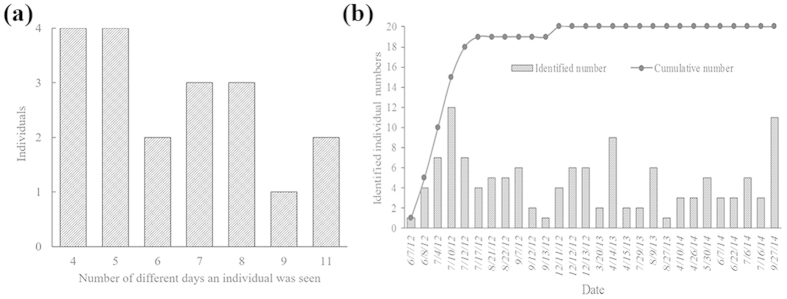
Frequency distribution of resightings of individual Indo-Pacific humpback dolphins (a) and identified individual numbers across the surveyed time (b).

Between 2012 and 2014, 69 line-transect vessel surveys were conducted in Shantou waters. A total of 51 schools of IPHBDs were sighted, from which only 19 IPHBDs were catalogued (excluding one calf). The identified animals included 18 adults and 1 spotted juvenile, with the exception of one calf. All of the individuals were photographed on more than four occasions, with two individuals photographed as many as 11 times (Fig. 5a). Overall, the rate of discovery (the cumulative number of identified individuals) reached a plateau before the end of the study period, suggesting that the vast majority of animals in the study area had been captured (Fig. 5b).

#### Site fidelity (SF)

**Figure 6:**
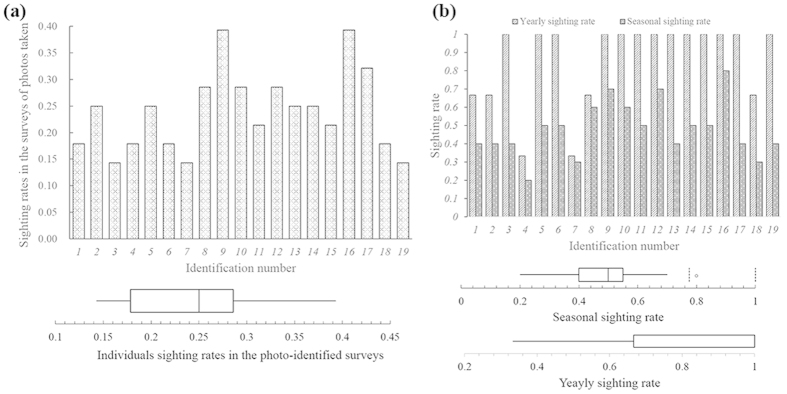
Site fidelity evaluations using individual sighting rates in the photo-marked surveys (a) and the seasonal and yearly sighting rates (b).

There were some variations in the sighting rate (mean 0.239 ± SE 0.017) of the 19 marked individuals, excluding calves, upon evaluation using the total photo-identification survey data (Fig. 6a). However, all of these individuals were sighted in more than 10% of surveys with photographs taken (minimum 14%), indicating moderate and high sighting rates. All of the individuals were sighted in more than two seasons (median = 0.5, 5 seasons), and one individual was sighted in 8 of the 10 surveyed seasons (Fig. 6b). Although seasonal sighting rates varied, no significant difference was found using the chi-square method (χ^2^ = 16.72, *df* = 18, p = 0.54). Additionally, yearly sighting rates (mean 0.86 ± SE 0.053, sightings per year) indicated that many of the IPHBDs identified were seen in more than two calendar years, and no significant variations were found from the overall statistical data (χ^2^ = 23.85, *df* = 18, p = 0.16).

#### Residence time (RT)

**Figure 7:**
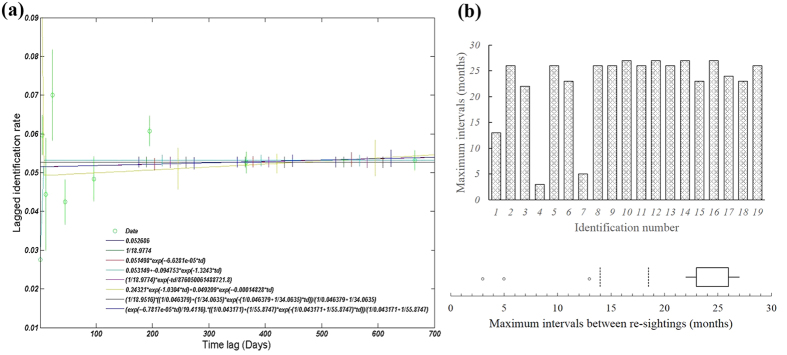
Computation of lagged identification rates (dots) for IPHBDs in Shantou waters using SOCPROG^50^, with vertical lines indicating jackknifed error bars (a) and maximum intervals of individual resighting (b).

Of the four pairs of models applied to the data using SOCPROG^50^, the “closed population” model curve matched the data best (AIC = 3119.4748, Table 1), although the accuracy of this model’s fit differed little from the emigration/mortality model (ΔAIC = 1.8634) and the emigration/reimmigration model (ΔAIC = 2.316) in comparison to the emigration + reimmigration + mortality model (ΔAIC = 21.7776) (Table 1). All models produced the same graphic display (straight line, Fig. 7a). Estimates of the mean population size and residence times from the closed model indicate that approximately 19 (18.9774 ± SE 0.48358; 95% CI = 18.0826, 19.9128) Chinese white dolphins were ordinarily resident in Shantou waters. The LIR (lagged identification rate) slightly fluctuated in short time lags, with relatively higher standard errors compared to the actual estimates (Fig. 7a), likely indicating that individuals exhibited a variable resighting pattern. However, the LIR remained stable over time, suggesting no influx or outflux. Further, the maximum intervals between the resightings of each IPHBD individual (mean 22.42 ± SE 1.66 month) indicated the long time spent in the study area (Fig. 7b).

## Discussion

The species extinction rate in the Anthropocene has accelerated significantly^51^,^52^, and the process of biodiversity loss has already transgressed the planetary boundaries^53^,^54^. The most important issue in the conservation of endangered species is efficiency, in terms of collecting the most information for effective conservation under the conditions of limited resources^55^,^56^. The population spatial structure and spreading ability influence population dynamics and genetic variation and thus are of basic importance to conservation biology^57^,^58^,^59^,^60^,^61^. Long-term and large-scale monitoring to investigate the spatial structure requires the substantial input of time and funds^62^. Monitoring the potential distributions and connections (migration or dispersal) of humpback dolphins along the large-scale coastline of the eastern Atlantic, Indian, and western Pacific Oceans (Fig. 1a) requires considerable effort and a large budget. Given increasing budgetary constraints, it is essential to find an effective way to minimize data redundancy and resource investment to achieve research targets. Here, we assessed the spatial patterns of humpback dolphins in eastern Guangdong in China for the first time, which is important for the regional risk evaluation of conspecific populations^26^,^63^,^64^. Additionally, as a case study, the survey pattern that we developed could be applied in a timely and economic manner to the spatial and temporal assessment in a large geographic range.

To substantiate the primary hypothesis that there is a certain amount of hitherto unknown IPHBD units in the estuarine waters between XM and PRE (more than 550 km) and to investigate the temporal and biogeographic patterns to inform the future conservation and management, we used the standard LEK interview method to develop empirical links between the spatial and temporal distributions of IPHBDs and implemented vessel-based surveys in Shantou waters to collect direct ecological monitoring data. These two methods complement each other, and the residence pattern estimates were consistent. In the interview survey module, our study provided the first quantitative evidence of the existence of IPHBDs in the estuaries of the region, historically and currently (Fig. 3). IPHBDs in these regions have declined in number, and some locations have undergone local population extinction since the 1970 s (e.g., Shenquan and Gangkou Town). The regional population extinction and the significant differences in sighting data from their neighboring sites demonstrate the spatial heterogeneity and disjunct distributions (or distribution gap). Further, the test of the seasonal sighting data collected through interviews showed seasonal distribution patterns (Fig. 4), indicating that each location had a similar four-season contribution and likely no seasonal preference and migration. In the boat-based survey module (1) the cumulative number of identified individuals reached a plateau in the first survey year, and the number did not change in the following 2 years (Fig. 5b), indicating no immigration to Shantou waters; (2) the IPHBD units in Shantou waters had moderate to high sighting rates and were present in multiple seasons and most were sighted in the 3 years during the survey (yearly sighting rate was 0.86) (Fig. 6), indicating that all individuals were permanent residents^65^,^66^; and (3) the lagged identification rate (Fig. 7a) estimation^67^ and individual maximum intervals testing demonstrated that the unit of IPHBDs in Shantou waters was a closed population with strong site fidelity and was isolated from conspecific populations along the China coast. Taken together, the data from LEK and the ecological monitoring suggest that current IPHBDs units in eastern Guangdong estuaries have the spatial structure of geographic isolation.

**Figure 8:**
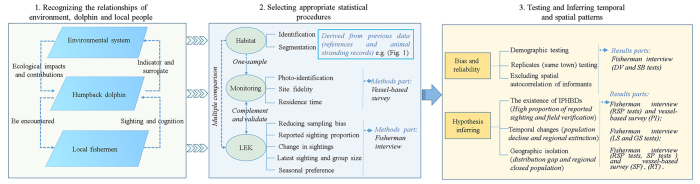
Framework of spatial and temporal pattern assessment.

Under the current circumstances, approaches and purposes to collect LEK vary markedly among studies. A standardized framework will help with the integration and interpretation of ecological knowledge. In our framework (Fig. 8), we first discussed the relationship of the three key components (environmental system, vulnerable or endangered species and local people) involved in the spatial and temporal assessment: (1) ecological impacts and contributions (both natural and anthropogenic) of habitat changes can influence dolphin’s ecology^3^,^20^,^25^,^26^; dolphins represent surrogates for wider biodiversity and act as indicators or sentinels of a complex ecosystem^16^,^17^,^18^,^19^,^68^,^69^; and (2) dolphins may be encountered relatively frequently, either incidentally or deliberately (through targeted exploitation), by local people utilizing the same habitats; local fishermen were exposed to different levels of species sightings and have different experiential knowledge (perception or cognition) of easily identifiable species and could provide useful information for conservation^19^,^46^,^48^. Second, data from the environment, dolphin and LEK were quantified, and appropriate statistical procedures were selected in the framework: (1) the habitat was identified and segmented into several patches according to the habitat use patterns of the humpback dolphin and was transformed to nominal data, which were used as independent variables or covariates in the spatial and temporal testing; (2) ecological monitoring (boat-based survey) was conducted in one of the patches, and the residence pattern (migratory or resident) parameters, such as sighting rate, site fidelity and residence time, were analyzed; and (3) multiple comparison (difference and correlation) was conducted on the patches based on the variables (demographic variables, reported sighting proportion, seasonal preference, latest sighting and group size). In this process, the biases were excluded, and reliability was tested. Finally, the hypothesis was inferred based on the significance tests of monitoring and LEK data in this study and previous spatial pattern theories^46^,^65^,^66^,^67^,^70^.

The question of whether there are connections of IPHBD units between XM and PRE has been brought into focus. However, the existing research focuses on genetic differentiation, and the results are controversial^40^,^41^. The data of the D-loop and mtDNA cytochrome *b* from the PRE (n = 6) and XM (n = 6) waters indicate that there is the possibility of gene exchange between these two units^40^. In contrast, the analysis of a 287-bp mitochondrial control region from 65 IPHBDs from these two regions (PRE n = 41, XM n = 24) demonstrates the highly significant genetic differentiation^41^. Another study based on the 332-bp mtDNA control region (PRE n = 33, XM n = 11) and microsatellite (PRE n = 15, XM, n = 8) showed no statistically significant differentiation^39^. These conflicting results suggest the deficient power of genetic methodology to assess the current isolation status of IPHBDs in China’s waters. Furthermore, the intrinsic features and biases generated from uncertain sample size, sampling sites, sampling gaps, genetic markers, animal generation time and genetic drift^71^,^72^ make it difficult to reach firm conclusions. Particularly, considering the character of endangered species (small size of population in some locations and insufficient number of stranded carcasses, which limited the sampling), and the risk of biopsy^73^, we do not think that genetic monitoring is a practical and optimal approach. Here, we proposed a framework to evaluate the connections between these two locations (IPHBD units in XM and PRE have been isolated, and between these two regions, there are some small residual populations). Our scenario is consistent with those of the photo identification, which, while not thoroughly analyzed, demonstrates that no individuals from the two populations (XM and PRE) have been found to mix^45^, and is consistent with the conclusions of recent ecological monitoring studies of the second largest population of humpback dolphins in Zhanjiang, China^31^. Comparing the photographs obtained to different catalogs of humpback dolphins from Chinese waters would further strengthen the conclusion. The temporal (local population decline and extinction) and spatial (biogeographic isolation) patterns in humpback dolphins likely represent the deterioration and fragmentation of the marine coastal ecosystem in China. Considering the potential Allee effect of the small population, great efforts are needed to achieve conservation and management. Given that coastal dolphins experience similar impacts from local ecosystems (both natural and anthropogenic factors), we argue that the spatial and temporal changes of IPHBDs in the Guangdong estuaries are likely to be an indicator of the fragmentation in distributions and decline in local populations of global humpback dolphins, and we hope that the loss of Baiji^74^ and the rapid decline of the Yangtze finless porpoise^75^,^76^,^77^ in China will increase concern and serve as a stern lesson for the habitat and species conservation of humpback dolphins.

Previous research has shown that interview surveys are useful and effective tools in conservation and ecology due to the time and cost effectiveness of the collection of ecological and non-ecological data on a large scale (wide geographic areas; both historical and current information)^46^,^47^,^48^,^78^,^79^,^80^. Fishermen spend a considerable proportion of their lives on the water, and they are familiar with local species, so their cumulative experiences sometimes provide more comprehensive information than short-term surveys^48^. Turvey and his colleagues concluded that the LEK method is a useful alternative monitoring approach for assessing the spatial and temporal extinction dynamics of fresh water cetaceans^46^. Sometimes age- or experience-related differences in perception (generational amnesia and personal amnesia) and biased responses (e.g., misidentification) by interviewees can contribute substantial error to surveys^81^,^82^,^83^. In our study, we minimized interviewer-related error (biased response) through standardized consulting (e.g., one-to-one interview; questions and answer choices of “closed questions” that were simple, unambiguous, and straightforward; detailed descriptions were encouraged in “open questions”) and optimized the interview design (e.g., Chaozhou [Teochew] speaking interviewers; using unprompted cue card; using other “test” cetaceans; requirement for key diagnostic information). We tested for sample bias in fishermen ages and years of fishing experience in each location, and no significant difference was detected. Additionally, our results indicated that there was no significant correlation between the sighting data sets and fishermen ages and years of fishing, excluding the possibility of the bias of generational amnesia. In addition, the replicate communities or docks in one estuary presented congruence (no evidence of a significant difference was found), rejecting the possibility of potential error and bias. We are confident that the reported sighting data in our survey are largely accurate. Both demographic tests and control group tests in each location prevent the bias of informants from spatial autocorrelation. Given the accelerating biodiversity loss rate and increasing budgetary constraints, we think our pattern is an effective way to clarify the spatial and temporal patterns of endangered species, particularly when population distributions are large-scale and traditional sampling methods are expensive and difficult to implement.

More generally, we developed a useful temporal and spatial identification framework based on a case study of locally endangered humpback dolphins. Using this pattern, we identified a small population in Shantou waters that had previously been neglected and supplied basic information of the spatial (disjunct distribution) and temporal (local population decline and regional extinction) dynamics of humpback dolphins between XM and PRE for the first time. The population changes are an indicator of or a prelude to the fragmentation of the distributions and decline in the local population of global humpback dolphins. Our framework, combining LEK evaluation and ecological monitoring, can serve as a flexible tool for regional and global efforts to identify spatial patterns of threatened coastal delphinid and to support further conservation and management, especially when no historical and spatial field data are available.

## Methods

Data were collected with approval from the Administration of Oceanic and Fishery of Guangdong Province. The methods were conducted in accordance with the approved guidelines of Guangdong Provincial Key Laboratory of Marine Biology, Shantou University. All of the informants were informed of the study and gave informed consent after a standardized interview.

### Fisherman interviews

#### Data collection

Interviews were conducted in fishing communities at 16 localities in 12 towns covering all the estuaries with possible IPHBD occurrences along the coastline between XM and PRE (Fig. 1b) during the fishing moratorium from June 1st to August 1st, 2013. The interview formats were designed using standard LEK interview techniques^46^,^47^,^48^,^78^,^80^,^84^. The informants were typically located in fishing settlements or the harbor and varied widely in their ages and fishing practices, which we consider to be broadly representative of the wider sample of fishers in each location, especially in terms of their relevant ecological knowledge and experience. As part of a wider series of interview questions, informants were asked about their age, number of years fishing at sea, the boundaries of where they went fishing, whether they had seen IPHBD, the last time, exact location and group size when that they saw IPHBD, the particular time and location of a year that they saw IPHBD, whether they considered that IPHBD had undergone a population decline, the time of the changes and the possible influence factors. Cue cards of photos (of live IPHBDs that we took in the field) and drawings (images of cetaceans from the published book “Chinese cetaceans”) of IPHBD and other cetaceans^85^ were shown to all informants to test their accurate identification of these species and the validity of their responses^48^,^78^. All informants were interviewed on a one-to-one basis in relaxed, informal settings by a native Chaozhou (Teochew) speaker who used a standard anonymous questionnaire containing descriptive, structured, and contrast questions.

#### Questionnaire analysis

To minimize the influence of sampling and implicit biases, the following were performed in the process of questionnaire analysis. (1) The questionnaire samples were chosen from informants that were not yet retired to ensure that clear and exact information of IPHBD current status was collected. Informant data were analyzed spatially according to the reported fishing distribution of each informant. (2) Special care was taken to verify the IPHBD sighting records, with informants required to provide detailed animal character information of their sighting (e.g., medium-sized, white or pink color, long and narrow rostrum). We did not regard information from the respondents as valid unless the description contained the key diagnostic characteristics of IPHBDs mentioned above. (3) The potential demographic differences between informant groups were tested by examining whether these geographical locations predicted differences in the age of informants and their years of fishing. (4) The potential demographic variation was further investigated by testing whether IPHBD sighting events (e.g., saw IPHBDs, recognized a decline in IPHBDs) in each location could be predicted by informants’ age or years of fishing. (5) To assess the reliability and validity of the interview, the conformance of the data from different sites in the same estuary was tested (Fig. 1b, 16 localities in 12 towns). For the locations that had recent records of IPHBD sightings, the data in the same estuary were merged, and one-way ANOVA and χ^2^ tests were performed on the latest-sighting records and seasonal sighting data to estimate whether current IPHBD units in these estuaries had the similar temporal distribution pattern.

We used a χ^2^ tests to evaluate whether the probabilities of IPHBD sightings of informants and the levels of each seasonal sighting were differentially distributed over levels of the locations. (1) The latest-sighting data sets (the medians and distribution of the latest-sighting time) in each local area were analyzed using nonparametric tests to evaluate the current biogeographic pattern of IPHBDs in each location. (2) The reported group size of the latest sightings in the past ten years was tested using variance analysis, and the data in adjacent locations were compared. Combined with the tests of the latest-sighting time, the current distribution pattern and potential spatial evolution pattern were evaluated. (3) The seasonal distribution of IPHBDs in each specific location was estimated from the season/month sighting data with a one-way analysis of variance. We used the frequencies ratio of IPHBD sightings in each season to measure the effect of season on IPHBD sighting; when a sighting event occurred at least one time in a season, we assigned “one” to the corresponding season, and to eliminate the influence of different sample sizes in each location, we used the frequency ratio instead of the records of each season in each location for the ANOVA analyses. For most of the fishermen who went fishing each day when weather and marine conditions were stable, we assumed that the potential confounding demographic variable ‘survey effort’ (by fishermen) was essentially continuous and contributed equally to the variation of the IPHBD encounter probability, which was uncorrelated with informant age or location of the fishing community across the region.

### Vessel-based survey

#### *Photo* identification

The boat-based survey area included the Hanjiang and Rongjiang estuaries (23°25’N–116°50’E) (Fig. 1c) and the coastal adjacent areas, covering an approximate area of 288.64 km^2^. Because IPHBDs are typically found in waters less than 20 m deep and up to 6 km offshore^35^,^43^,^44^, the outside boundary of our survey was approximately 10 km offshore. Exploratory surveys were conducted at a steady cruising speed of 8–12 km/hr under a sea state of Beaufort scale (BF) <4, from a 14 m long fishing boat, powered by a 72 hp engine, when the weather and marine conditions were stable. Survey routes were designed with zigzag transect lines, and each route began at a different site to ensure that multiple areas of the study site were visited with consistent frequency. Surveys were interrupted to observe, photograph and record dolphins. The survey boat slowed and followed their movements for a minimum of 30 minutes. To avoid disturbing the animals, a distance of at least 30 meters was maintained at all times. Photographs of individuals were taken from the animals’ side, perpendicular to the body axis, and concentrated mainly on the dorsal fin. Individual identifications were based mostly on shape, size and the position of nicks and marks on the dorsal fin, supplemented by variation in pigment patterns and body scratches. Only good photos (i.e., sharp focus, good angle and non-reflection on the dolphin’s body) were used for photo identification analyses^70^,^86^,^87^. The positions of dolphin group sightings were collected using a handheld Geographic Positioning System unit (Garmin eTrex Venture GPS, Olathe, KS USA). We defined the term “school” or “group” as dolphins that were spatially close (all dolphins within a 100 m radius of each other) and involved in similar activities^70^. To minimize the likelihood of dependence in the data, the first sighting of the day, even though some animals were identified more than once in a day, was used in the analyses.

#### Site fidelity

Site fidelity was defined as the tendency of an individual IPHBD to occupy the studied area according to previous studies^88^,^89^. To assess whether the dolphins are residents, transients or occasional visitors, the site fidelity patterns of individually identified dolphins were investigated using a previous evaluation method that was applied to *Tursiops aduncus* population surveys^65^,^66^. According to the sighting rates and presence across seasons, residents (R) were defined as dolphins with moderate (sighted between 10 ~ 30% of surveys with photographs taken) and high sighting rates (sighted more than 30%) and present in multiple seasons. Transients (T) were defined as animals with low sighting rates (sighted in less than 10%) and present in only one season, whereas occasional visitors (OV) were defined as those with low sighting rates but present in multiple seasons^65^,^66^. In addition, we used the seasonal and yearly sighting rate to investigate the presence of identified individuals in the study area over time according to the method used in the residence patterns analysis of Australian snubfin and humpback dolphins^70^. We calculated the number of seasons and calendar years of an identified dolphin as a proportion of the total surveys in which at least one survey was conducted. Potentially, seasonal sighting rates ranged between 0.1 (i.e., animals sighted in only one season out of 10) and 1.0 (an individual sighted in all seasons). Similarly, potential yearly sighting rates ranged from 0.3 (i.e., animals sighted in only one year out of 3) to 1.0 for an individual sighted in all three years of the study. Chi-square analysis was used to compare the proportion of individuals with different sighting rates to evaluate the site fidelity of an individual IPHBD in Shantou waters.

#### Residence times

Randomly collected animal individual-identification data in one location could supply essential information about the lagged identification rate (LIR) as well as residence times inside and outside the study area^67^. We can analyze animals’ movements by calculating the probability that an individual is re-identified in the specific area after a certain time lag: (1) some animals emigrated to the study area or died with time log; (2) some animals were resident; and/or (3) other animals immigrated to or cyclically visited the study area with time lag^67^. This study estimated the temporal use of Shantou waters by individual IPHBD based on plots of LIR against time lag using the software SOCPROG^50^. The best fitting models were selected from the models of closed, emigration/mortality and emigration + reimmigration (+mortality) using the Akaike Information Criterion (AIC) or quasi-AIC (QAIC)^67^,^90^. Jackknife techniques were used to calculate the 95% confidence intervals and standard errors for each model parameter. Further, to investigate the potential amount of time (residence time) that an individual IPHBD spent in the study area during the investigation, the maximum monthly intervals between captures of each individual were calculated.

## Additional Information

**How to cite this article**: Jingzhen, W. *et al.* A framework for the assessment of the spatial and temporal patterns of threatened coastal delphinids. *Sci. Rep.* **6**, 19883; doi: 10.1038/srep19883 (2016).

## Supplementary Material

Supplementary Information

## Figures and Tables

**Table 1:** Analysis of models that can be fitted to LIRs (lagged identification rates) for humpback dolphins identified in Shantou waters using SOCPROG^50^.

Model	AIC value	Maximum-likelihood value for parameters
Closed	3119.4748	N = 18.9774 ± 0.48358
Emigration/Mortality	3121.3382	N = 18.9774 ± 0.54259
		Mean residence time = 876050061488721.8 ± 665625241746216.4
Emigration + Reimmigration	3121.7908	N = 18.9516 ± 0.87426
		Mean time in study area = 34.0635 ± 19.7447
		Mean time out of study area = 0.046379 ± 5.165
Emigration + Reimmigration +Mortality	3141.2524	N = 19.4116 ± 1.1469
		Mean time in study area = 55.8747 ± 28.9441
		Mean time out of study area = 0.043171 ± 5.6489
		Mortality rate = −6.7817e-05 ± 0.00011125

(Using AIC rather than QAIC due to no sign of overdispersion).
