# Supplementary material for: A framework for the assessment of the spatial and temporal patterns of threatened coastal delphinids
Source: Sci Rep. 2016 Jan 25;6:19883. doi: 10.1038/srep19883 (PMC4726438; doi:10.1038/srep19883)
Supplement: Supplementary Information [file srep19883-s1.pdf]

A framework for the assessment of the spatial and temporal patterns of threatened coastal delphinids

Wang Jingzhen[1,2], Yang Yingting[1], Yang Feng[1], Li Yuelin[1], Li Lianjie[1], Lin Derun[1], He Tangtian[3], Liang Bo[4], Zhang Tao[1], Lin Yao[1], Li Ping[1], Liu Wenhua[1*]

1 Marine Biology Institute, Shantou University, Guangdong 515063, China

2 College of Ocean, Qinzhou University, Guangxi 535000, China

3 Department of Biology and Chemistry, City University of Hong Kong

4 The Chinese University of Hong Kong

*To whom correspondence should be addressed:

Wenhua Liu, Ph.D., Shantou University, Shantou, Guangdong 515063, P. R. China. Fax: +86-754-86500614; E-mail: whliu@stu.edu.cn

Contents

Eastern Guangdong Fishermen Survey Questionnaire (Chinese white dolphin and other cetaceans);

Eastern Guangdong Fishermen Survey Questionnaire

(Chinese white dolphin and other cetaceans)

Location: _____ Serial No.: _____ Interviewer: _____ Time: _____

Information of the informant: Gender: _____ Age: _____ Status: _____ Retired (Y/N): _____
Information of the vessels of the informant: Horsepower: _____ Length: _____

**Informant is asked to identify a Chinese white dolphin (CWD) or the other cetacean from a series of photographs without being prompted. If he/she is able to do this, the following questions are asked.

1, How many years have you been fishing?

2, Which area do you usually go fishing?

3, Do you know what a CWD is? (Y/N)

Have you ever seen a CWD in your lifetime? (Y/N)

What do you call it? Local name: _____

4, When and where is the last time you saw a CWD group and how many CWD did you see?

Time: _____ Location: _____ Number of individuals: _____

5, Can you recall some sighting cases of CWD and other cetaceans in the past few years (time and location)? Can you describe what you had seen? (population number and individual characteristics)

Cetaceans	Location	Particular Time (M/Y or seasons/Y)	Population number	Characteristic description
1 CWD				

Note: Characteristic description: e.g. length, color or pattern, with or without fin and snout，long or short snout, extended forehead, with or without spout.

6, Do you see more CWD or other cetaceans at a particular season or month of a year? (Y/N) and if so then which season or month? What is the outer environment like when you see a CWD? (Weather, tide, and wave amplitude)

Cetaceans	1 CWD	Cetacean 2_______	Cetacean 3________	Cetacean 4________
Month or season				
Weather				
Tide				
Wave				
Other conditions				
Option sets	Tide: A, rising tide B, falling tide C, high tide D, low tide E, irrelevant<br>Weather: A, sunny B, cloudy C, rainy D, moderate rain E irrelevant<br>Seasons (months): A, Spring (2, 3, 4) B, Summer (5, 6, 7) C, Autumn (8, 9, 10)<br>D, Winter (11,12,1) E, Most of the year or not specific seasons<br>Wave: A, quiet B, moderate C, stormy			
Note				

7, How has the population of CWD and other cetaceans changed in these past few years? (A increase B decrease C constant) When (any particular period) and why did this change happen?

Particular periods	A 1970s	B 1980s	C 1990s	D 2000s	E 2010s
Population changes					
Changing reasons					
Related events					
Option sets	1, water pollution 2, increasing fishing vessel 3, overharvesting 4, coastal industry (dam building, sand mining, coastal factory) 5, direct injury 6, other reasons _____				
**Note (other cetaceans)**					

8, Have the rivers nearby gone through any large changes in the past few years (runoff volume changes etc.)? (Y/N) Can you supply some details of the changes? (river name, what changes, changed time, potential causes)

River name	Changes	Changed time	Causes

9, Can you remember any cases of CWD stranding or death? (Y/N)

Describe any related events that were seen (location, date, other details)

Time (M/Y, Season/Y)	Location	Cetacean species	Handling way	Other details
		CWD		

10, Do you know any events of accidentally capture of cetacean? What kind of fishing gear? When and where did this happened? How did the people deal with these cetaceans.

Cetacean	Fishing gears	Season or month	Location	Dead or alive	
1 CWD					
Fishing gears	A Gill net B Stake-net C Drag nets D Rolling hooks E Electro-fishing<br>F Other type of fishing gear (describe)_______				

11, Do you know whom to contact to when encountering a stranded dolphin? (Y/N)

Contacts: _______

12, Do you know that CWD is the first-class state protected animal? (Y/N)

Has the local government promoted on this (e.g. propaganda or education work)? (Y/N)

How is CWD related to your life?

Does it have any specific meanings in your culture?
